# Depressive and Sexual Disorders during the First and Second Wave of the COVID-19 Pandemic among Young Polish Women

**DOI:** 10.3390/ijerph19031887

**Published:** 2022-02-08

**Authors:** Ewa Szuster, Paulina Kostrzewska, Anna Pawlikowska, Amanda Mandera, Małgorzata Biernikiewicz, Małgorzata Sobieszczańska, Krystyna Rożek-Piechura, Grażyna Jarząbek-Bielecka, Agnieszka Rusiecka, Dariusz Kałka

**Affiliations:** 1 Cardiosexology Students Club, Wroclaw Medical University, 50-368 Wrocław, Poland; ewa.szuster8@gmail.com (E.S.); pkostrzewska1@gmail.com (P.K.); anna.pawlikowska96@gmail.com (A.P.); amanda.mandera@vp.pl (A.M.); 2 Studio Słowa, 50-357 Wrocław, Poland; malgorzata@biernikiewicz.pl; 3 Clinical Department of Geriatrics, Wroclaw Medical University, 50-369 Wrocław, Poland; malsobie100@gmail.com; 4 Faculty of Physiotherapy, Wroclaw University of Health and Sport Sciences, 51-612 Wrocław, Poland; krystyna.rozek-piechura@awf.wroc.pl; 5 Department of Perinatology and Gynecology, Poznan University of Medical Sciences, 60-535 Poznań, Poland; grajarz@tlen.pl; 6 Statistical Analysis Centre, Wroclaw Medical University, 50-367 Wrocław, Poland; agnieszka.rusiecka@umw.edu.pl; 7 Men’s Health Centre in Wrocław, 53-151 Wroclaw, Poland

**Keywords:** COVID-19 pandemic, women, health, sexual functioning, mental health, Polish population, online survey

## Abstract

We investigated whether long-term social restrictions and COVID-19 exposure have different impacts on the mental and sexual health of Polish women compared to the effects experienced at the beginning of the pandemic. An online survey was conducted among Polish women via Facebook groups. The Beck Depression Inventory (BDI) and Female Sexual Function Index (FSFI) scores were compared for the first wave (April–May 2020) and the second wave (November 2020 to February 2021) of the pandemic. We enrolled 1644 participants (mean age 25.11 ± 7.09 years) during the first wave and 720 participants (mean age 23.23 ± 5.34 years) during the second wave of COVID-19 pandemic. Significant differences were observed in libido levels and frequency of sexual activity before and during the first and second wave of the COVID-19 pandemic (both *p* < 0.001). The percentage of participants under psychiatric or psychological care increased from 6.5% to 14.44% and those who were anxious about the health conditions of loved ones increased from 57.5% to 65.14%. BDI scores increased significantly from 11 (IQR 5–18) to 12 (IQR 7–20). The change in the FSFI score was not significant (27.01 ± 7.61 vs. 26.38 ± 7.76). The COVID-19 pandemic affected various aspects of human life, including sexual life. The data obtained during the first and the second wave of the COVID-19 pandemic in Poland showed that female sexual dysfunction did not differ, but depressive symptoms and fear intensified.

## 1. Introduction

Coronavirus belongs to the group of RNA viruses that directly affect the functioning of the respiratory system, but can also affect the work of other organs, e.g., the heart or central nervous system. The first case of coronavirus was diagnosed in Poland on 4 March 2020. On the 11th of March, the restrictions in Poland began. Schools, kindergartens and universities were closed. Then, restaurants and pubs followed. From the 25th of that month, everybody had to stay at home; only going out for shopping was possible. From the 3rd of April, forests and parks were closed. The 20th of April was the first day when restrictions were lifted. The next limitation started with the second wave—on the 16th of November, gyms and swimming pools were closed, then e-learning in schools was provided. From the 28th of December to the 17th of January, a national quarantine was implemented. In January, the lifting of restrictions began [1].

The COVID-19 virus was spreading rapidly and a few days later (on 11 March 2020), the WHO called this situation the COVID-19 pandemic, which was identified as the greatest health threat of the past century [2,3]. Since then, governments, scientists and public health organizations have been focusing on the coronavirus’s spread [4], prevention and vaccination. Across the world, the SARS-CoV-2 virus caused almost 5 million deaths [5]. Due to the rapid spread of COVID-19, public health authorities in many countries decided to provide numerous restrictions including travel bans, restrictions of social gatherings and closures of public schools [6]. This resulted in a global economic crisis and a deterioration in the mental health of the general population, particularly affecting medical personnel [7,8].

Attempts were also made to control the global pandemic by using masks in public areas, paying attention to personal hygiene practices, following social distancing rules and ensuring the isolation of sick individuals [9]. Social isolation unavoidably impacted the economy and functioning of society [6]. Furthermore, it was also connected with anxiety and widespread fear, which can lead to situations that negatively affect quality of life, including the development of depression and sexual dysfunction [10]. In Nowak et al.’s research, Poles demonstrated varying fear levels regarding different aspects of the COVID-19 pandemic and its potential consequences. The highest ranked was the fear over the health of relatives, followed by fear of pandemic-induced economic crises, fear regarding the use of the pandemic to control citizens, fears around individuals’ own health, as well as fears of pandemic-induced political crises, and of job losses. Women displayed greater fear regarding every considered aspect [11]. Idzik et al. also revealed anxiety and depression disorders in Polish women. Two in three women experienced loneliness. Women aged 18–29 years showed the highest levels of anxiety, depression, irritability, and loneliness [12].

Sexual health is one of the factors influencing human well-being. Although sexual rights must be protected in times of crisis, this is often overlooked in the face of more pressing problems. The New York City Department of Health issued recommendations regarding sexual activity during the COVID-19 pandemic to minimize the risk of virus transmission. It was considered the safest practice was to masturbate and the second safest practice was having sex with a cohabitating partner [13]. The frequency of masturbation behavior, and the use of sex toys and pornography increased [14].

Many scientists investigating mental health found a higher prevalence of depression, anxiety, insomnia and violence in the population during the outbreak of COVID-19, concluding that the pandemic harmed the mental health of the population [15,16]. Reports from the literature suggest a link between women’s quality of life, sexual health and stressful events in their life [17,18]. The evidence on the impact of the COVID-19 pandemic on sexual behavior is growing [19]. Our previous study, which we performed at the beginning of the pandemic (April–May 2020) revealed that the COVID-19 lockdown setting was associated with a high occurrence of depressive symptoms and increased risk of sexual dysfunction in women, translating into a decreased libido and a lower frequency of sexual activity [20]. The aim of this study was to investigate the frequency of depressive symptoms and changes in sexual functioning in Polish women during long-term social restrictions and COVID-19 exposure using subjective, patient-centric measures. To this end, we evaluated correlations between BDI and FSFI scores as well as psychological characteristics.

## 2. Materials and Methods

This was a cross-sectional study that was repeated twice during the COVID-19 pandemic. The data on the first wave of the COVID-19 pandemic were collected between 22 April 2020 and 7 May 2020. The study on the second wave of the pandemic was conducted from 4 November 2020 to 14 February 2021. Invitations with links to the online questionnaire, which used Google Forms, were shared in Facebook groups containing female users of all ages. Two different groups of women were administered the same surveys during the first and second pandemic waves. Participation was voluntary and the respondents were informed about the aim of the study and were asked to read and complete consent forms. Each woman was asked to complete the Beck Depression Inventory (BDI) and Female Sexual Function Index (FSFI) questionnaires. The forms also included demographic questions and questions about women’s behavior and feelings during the pandemic. For the analysis, we included women of 18 years or older, those who were sexually active as well those who returned fully completed questionnaires. The respondents were asked to read information about the aim of the study and to accept the rules of the research before completing the questionnaire. They were informed that they could ask questions and that participation was voluntary. The study was approved by the Commission of Bioethics at Wroclaw Medical University, Wrocław, Poland (KB-424/2021).

The FSFI is a validated questionnaire that consists of 19 questions, which are classified into 6 sections—desire, arousal, lubrication, orgasm, satisfaction and pain or discomfort [21]. Each section is scored from 1 to 6 points and the maximum score is 36 points. Our version was validated for the Polish population by Nowosielski et al. with the optimal cut-off score of 27.50 [22]. Scores below 27.50 indicate sexual dysfunction.

The BDI is one of the most popular questionnaires used to measure depressive syndromes, and it consists of a 21-question multiple-choice self-report inventory. Each question is scored from 0 to 3. Higher total scores are correlated with more severe depressive symptoms, which indicate minimal (0–11 points), mild (12−19), moderate (20–25) or severe (26–63) depression [23].

The collected data were statistically analyzed with Statistica software v. 13.3 (StatSoft, Tulsa, OK, USA). Descriptive data were presented as numbers and percentages for categorical variables, and as the mean, standard deviation, median, range and interquartile range for numerical variables. The distribution of continuous variables was tested using visual (histogram) and analytical methods (Kolmogorov–Smirnov/Shapiro–Wilk tests). The Chi-square test or Fisher exact test were used for the comparison of qualitative variables (BDI scores and psychological characteristics). The Mann–Whitney U test was used for the subgroup analysis of non-normally distributed variables and a Student’s *t*-test was used for the comparison of means for normally distributed data. The Pearson correlation coefficient with the Fisher Z-transformation was used to measure the association between FSFI, BDI and questions about the pandemic. We compared levels of FSFI and BDI during two waves of the pandemic using general linear models (GLM) to take into account the possible influence of socioeconomic covariates. We separately modelled the dependency of FSFI and BDI scores on a set of predictors including age as a continuous variable and four nominal predictors: wave of the pandemic (I or II), education (higher, secondary, lower), place of living (as in Appendix A, Table A1) and employment status (student, employed, working remotely, unemployed, sick leave). The best models were selected in a step procedure based on the Akaike Information Criterion (AIC). The sample size was calculated using the G* power package (Heinrich Heine University Dusseldorf; North Rhine-Westphalia; Germany). The differences were considered statistically significant at *p* < 0.05.

## 3. Results

Two study samples were analyzed. A total of 1644 participants during the first wave of the COVID-19 pandemic and 720 participants during the second wave of the COVID-19 pandemic were included in the study. The mean age of the first wave study population was 25.11 ± 7.09 years with a median age of 23 years, and the second wave study population had a mean age of 23.23 ± 5.34 years with a median of 23 years. In both waves, people with a higher education dominated. In addition, in both groups, students constituted the highest percentage of respondents. Most of them lived with a partner and in a big city. A summary of the characteristics of the study groups is presented in Appendix A, Table A1. Regarding the COVID-19 related characteristics, more people were in quarantine, had a history of infection with COVID-19, were currently infected, and had more friends and relatives affected by the disease. The COVID-19 related characteristics are presented in Appendix A, Table A2.

Regarding psychological condition, significantly more people confirmed being under psychiatric/psychological care during the second wave of the COVID-19 pandemic (6.5% vs. 14.44%; *p* < 0.001), but they felt significantly less isolated (*p* < 0.001). During the second wave of the COVID-19 pandemic, as compared to the first wave, more respondents strongly agreed or agreed that fear of the health condition of loved ones was a source of stress and depressed mood (n = 469; 65.14%). The results of the psychological characteristics of the study participants are presented in Appendix A, Table A3.

There was a statistically significant difference between the libido level before and during the first and second wave of the COVID-19 pandemic (*p* < 0.001), as well as a difference in the frequency of sexual activity before and during the first and second wave (*p* < 0.001). The results are presented in Table 1.

The best model explaining the BDI score indicated a strong influence of socioeconomic covariates (age: Wald stat. = 53.379, df = 1, *p* < 0.01; education: Wald stat. = 26.989, df = 2, *p* < 0.01; employment: Wald stat. = 9.204, df = 4, *p* = 0.05) but also included the wave of the pandemic (Wald stat. = 2.990, df = 1, *p* = 0.08). During the second wave, the BDI total score for all participants was 12 (IQR 7-20), which corresponds to the classification of mild depression. The results obtained from the first wave showed that total BDI score was 11, which corresponds to minimal depression. A direct comparison of BDI scores between the study group from the first and another group from second wave of the COVID-19 pandemic showed a higher percentage of participants with depressive disorders (mild, moderate and severe depression) in the second wave. Additionally, during the first wave of the pandemic, there were no significant differences in the number of BDI total scores between the groups of participants not subject to quarantine and those in quarantine (*p* = 0.41). However, during the second wave, the mean BDI score was significantly higher in quarantined participants (*p* = 0.044). The detailed results of the BDI questionnaire are presented in Table 2. The mean FSFI total score for second wave participants was 26.38 ± 7.76 (range 1.2–36), which was similar to that of the first wave, where the total FSFI score was 27.01 ± 7.61 (range 2–36). The final model that explained the FSFI score included education, place of living and employment status but not the wave of the pandemic. The detailed results of the FSFI scores are presented in Table 3. Correlations between BDI and FSFI scores as well as the psychological characteristics of the study groups were similar during both waves of the pandemic (Appendix A, Table A4).

## 4. Discussion

In March 2020, a statement calling upon governments and global health institutions to collect the sex and gender effects of the COVID-19 outbreak was published. The authors paid attention to the fact that during past outbreaks, the problem of gender-related consequences of epidemics was usually marginalized. It was also noticed that the degree to which disease outbreaks have a differential impact on women and men is the basis for understanding the primary and secondary health risks for various individuals and communities, and for developing effective and fair policies and interventions [24]. Lockdown and self-isolation at the beginning of the pandemic were connected with fear and anxiety, although the impact of long-term social isolation is still unknown. Many outbreaks that were provided in Poland revealed a higher prevalence of anxiety and depressive disorders, especially in Polish women [11,12,25].

The aim of this study was to investigate the difference in the occurrence of depressive symptoms and sexual function in women during the first and second waves of the pandemic. The results demonstrate that the difficult experience of the COVID-19 pandemic was associated with the higher occurrence of depressive symptoms and changes in sexual function during both waves of the pandemic.

Comparing the BDI results during the first and second wave among people who were quarantined, there is a clear difference, namely that with the duration of the pandemic, depressive symptoms intensified [26]. Moreover, Schuch and co-authors also noted an increased tendency of episodes of depression and anxiety in people who self-isolated and did not engage in any physical activity [27]. Many authors (e.g., Bhambhvani et al. [28], Ilgen et al. [19], Güzel and Döndü [9] and Fuchs et al. [29]) reported that the FSFI score decreased during the pandemic in comparison to the FSFI scores obtained before the pandemic. Our study showed no statistically significant difference in the FSFI score (27.01 vs. 26.38; *p* = 0.127) between waves of the pandemic. There is no study comparing the FSFI scores between waves of COVID-19. In comparison to other authors who investigated female sexual functioning in different countries, at different times of the pandemic, our results were similar (Bhambhvani et al. [28]—27.22 and Fuchs et al. [29]—25.8). The important fact is that our respondents were young women. Young people are less vulnerable to developing sexual dysfunctions. During the lockdown, due to remote education and working, people spent more time with a partner, which created more opportunities for sexual intercourse.

The COVID-19 pandemic is a significantly stressful factor that affects the health system, the economy and relationships. Its impact on these major aspects of everyday life could cause mental disorders, namely depression. Our findings confirm those of Ilgen et al. [19], who concluded that depression and anxiety increased during the pandemic. In their study as well as in our results, the mean score of BDI was classified as mild depression.

Stress is one of the major factors affecting sexual function, especially sexual desire. However, there are studies with varying results on this issue. The study conducted by Hall et al. concluded that women have better sexual activity during stressful times, which may be associated with spending more time with their partners [30]. Our findings depicting that chronic stress associated with the COVID-19 pandemic negatively affects women’s sexual life were confirmed by Yuksel et al. [31], who showed that the pandemic caused a significant deterioration in female sexual function during its duration. However, they found that despite the decreased quality of sexual life during the pandemic, the frequency of sexual intercourse increased.

The relationship between female sexual dysfunction and the presence of depression symptoms has been widely studied. Cohen et al. [32] showed a higher prevalence of sexual dysfunction in people with depressive disorders. Ilgen et al. [19] showed that the anxiety levels and BDI score significantly increased during the pandemic. However, the FSFI scores did not decrease. In addition, no correlation was found between FSFI and BDI scores. In our study, an inversely proportional relationship between BDI and FSFI was found, but the strength of the correlation was weak.

There are many guidelines and recommendations on preventing COVID-19 infection, such as self-isolation, using masks and disinfecting hands. The most efficient one is vaccination [33]. No vaccine works for every patient. Although the reducing of the infection or mortality rate depends on factors related to the specimen, there are also many patient-related aspects. Madison et al. described that distress may be a negative predictor of responses to vaccination [34]. Furthermore, psychological factors could worsen the vaccine’s efficiency as well as its side effects [35]. Our study was conducted before the introduction of the vaccine for everyone in Poland. The depressive disorders in our respondents could affect their immune responses to further vaccinations. Rapid diagnosis and treatment of mental disorders could prevent depressive disorders and improve immune responses to vaccination.

With the development of the pandemic, strict restrictions have been introduced in the world in order to inhibit person-to-person contact. Understandably, doctors’ attention was focused mainly on the symptoms caused by COVID-19, but it should not be forgotten that this new situation affects various aspects of human life, including sexual life. People are more likely to suffer from depression and loneliness while staying at home. Social campaigns informing people on how to look after their mental health, and encouraging the use of psychological consultations, should be considered. Furthermore, during the lockdown, participation in online meetings with friends and family, as well as webinars and other online activities, should be recommended. Local authorities should pay attention to popularizing outdoor sports activities that are safe during the pandemic and lockdown. These additional disorders are worth paying attention to, because fighting them is just as important as fighting the disease, and the long-term consequences can be serious.

**Figure A1:**
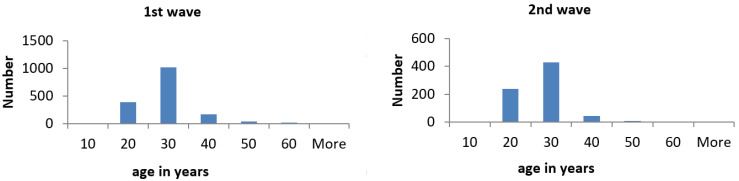
Age difference/distribution and sample size calculation.

It should be taken into consideration that the present study has some limitations. The main one is that Facebook groups are mostly used by young people, which was confirmed by the mean age (25.11 ± 7.09 years old—first wave; 23.23 ± 5.34 years old—second wave) of our study group. Moreover, computer and internet use is still not common among older age groups, so it is important to consider that our findings are not representative of the whole population of women. Our respondents were mostly high school or university students, which also could have affected sexual functioning. We also have to highlight the fact that despite the survey being distributed in the same way during the first and the second wave of the pandemic, there were differences between the enrolled groups. Our regression models showed an influence of socioeconomic covariates on the BDI and FSFI scores. The strong impact of socioeconomic status on sexual functioning was also reported by other researchers [36], while age differences do not seem to be clinically significant (Appendix A Figure A1). In addition, naturally, there were differences in terms of COVID-19 contact characteristics because more people were infected or had more infected friends and relatives. Many other factors may have an impact on women’s sexual functioning such as living with a partner, changes in the amount of time partners spend together, the presence of children and changes in the amount of time spent with them, relationship status, and the amount of stress resulting from these changes. Personal strategies of coping with stressful situations could also influence depressive disorders. Furthermore, male sexual behavior may affect female sexual functioning.

Prolonged lockdown followed by subsequent quarantines caused by further outbreaks of infections caused by the SARS-CoV-2 virus, as shown in this study, had a global negative impact on the depression symptoms and sexual function of the population of young women. Deterioration of mental health and sexual functioning may have potentially long-term negative multidimensional effects on the functioning of an individual. On top of this, many women had additional burdens during the pandemic, such as supervising their children during homeschooling and simultaneous remote working, that were not studied here. This should prompt the government, family physicians, sexologists and mental health counsellors to pay more attention to individuals who present with depressive symptoms and disorders of sexual functioning and to develop preventive and intervention measures to alleviate negative overall health effects.

## 5. Conclusions

Data obtained during the first and the second wave of the COVID-19 pandemic in Poland showed that female sexual dysfunction did not differ; however, depressive symptoms and fear intensified. The prolonged lockdown greatly increased perceived feelings of depression and loneliness in women in comparison to the first wave.

## Figures and Tables

**Table 1:** Sexual status characteristics of the study group during the first and second wave of the COVID-19 pandemic.

Variable	First Wave (*N* = 1644)		Second Wave (*N* = 720)	
Frequency of sexual activity before/during pandemic	I wave *p* < 0.001		II wave *p* = 0.028	
Several times a day	27 (1.6%)	36 (2.2%)	17 (2.36%)	19 (2.64%)
Every day	93 (5.7%)	84 (5.1%)	32 (4.44%)	37 (5.14%)
Several times a week	749 (45.6%)	579 (35.2%)	326 (45.28%)	260 (36.11%)
Once a week	221 (13.4%)	228 (13.9%)	99 (13.75%)	107 (14.86%)
Several times a month	325 (19.8%)	320 (19.5%)	132 (18.34%)	151 (20.97%)
Once a moth	55 (3.3%)	84 (5.1%)	23 (3.19%)	36 (5.00%)
Fewer than once a month	174 (10.6%)	313 (19.0%)	91 (12.64%)	110 (15.28%)
Libido level before/during pandemic	*p* < 0.001		*p* < 0.001	
High	521 (31.7%)	504 (30.7%)	234 (32.50%)	228 (31.67%)
Moderate	909 (55.3%)	747 (45.4%)	407 (56.53%)	320 (44.44%)
Decreased libido	214 (13.0%)	393 (23.9%)	79 (10.97%)	172 (23.89%)

**Table 2:** Beck Depression Inventory score during the first and second wave of the COVID-19 pandemic.

	First Wave, *N* = 1644	Second Wave, *N* = 720	
Total score			
median	11	12	
range	0–51	0–55	
IQR	5–18	7–20	
minimal depression—0–11 scores	858 (52.2%)	328 (45.55%)	
mild depression—12–19 scores	437 (26.6%)	211 (29.31%)	*p* = 0.024
moderate depression—20–25 scores	183 (11.1%)	91 (12.64%)	
severe depression—26–63 scores	166 (10.1%)	90 (12.50%)	

**Table 3:** Female Sexual Function Index score during the first and second wave of the COVID-19 pandemic.

Domain	Score, Mean ± SD		Range	
	I Wave	II Wave	I Wave	II Wave
Desire	4.16 ± 1.17	4.05 ± 1.19	1.2–6	1.2–6
Arousal	4.60 ± 1.52	4.54 ± 1.49	0–6	0–6
Lubrication	4.90± 1.60	4.88± 1.58	0–6	0–6
Orgasm	4.32 ± 1.69	4.17 ± 1.73	0–6	0–6
Satisfaction	4.53 ± 1.55	4.53 ± 1.55	0–6	0–6
Pain	4.49 ± 1.75	4.48 ± 1.74	0–6	0–6
Overall score	27.01 ± 7.61	26.38 ± 7.76	2–36	1.2–36

## Data Availability

Data are contained within the article.

## Appendix A

**Table A1:** Study group characteristics during the first and second wave of the COVID-19 pandemic.

Variable	First Wave (*N* = 1644)	Second Wave (*N* = 720)	*p*-Value
Age, years (distribution other than normal)	Mean 25.11 ± 7.09	Mean 23.23 ± 5.34	<0.001
	Median 23	Median 22.00	
	IQR (21–27)	IQR (18–25)	
	Range (18–67)	Range (18–55)	
Education			
Primary	88 (5.4%)	42 (5.83%)	<0.001
Vocational	74 (4.5%)	15 (2.08%)	
Secondary	780 (47.4%)	414 (57.5%)	
Higher	702 (42.7%)	249 (34.58%)	
Employment status			
Employed—working at work place	407 (24.8%)	165 (22.92%)	<0.001
Remote work	331 (20.1%)	66 (9.17%)	
Employment issues due to pandemic	55 (3.3%)	4 (0.56%)	
Sick leave	60 (3.6%)	19 (2.64%)	
Unemployed due to other reasons	58 (3.5%)	20 (2.78%)	
Full-time student	637 (38.8%)	435 (60.42%), including: students 391(54.31%) and pupils 44 (6.11%).	
Student—income lost	24 (1.5%)	2 (0.28%)	
Maternity leave/stay-at-home-mum	58 (3.5%)	5 (0.69%)	
Childcare due to COVID-19 pandemic	9 (0.5%)	1 (0.14%)	
Pensioner	5 (0.3%)	3 (0.42%)	
Marital status			
Single	278 (16.9%)	150 (20.84%)	<0.001
Married	302 (18.4%)	79 (10.97%)	
In partnership	1064 (64.7%)	491 (68.19%)	
Place of living			
Rural area	311 (18.9%)	139 (19.30%)	<0.001
City >50,000 inhabitants	267 (16.2%)	99 (13.75%)	
City from 50,000 to 100,000	142 (8.6%)	73 (1 0.14%)	
City from 100,000 to 250,000	186 (11.3%)	82 (11.39%)	
City above 250,000 inhabitants	738 (44.9%)	327 (45.42%)	

**Table A2:** COVID-19 related study group characteristics during the first and second wave of the COVID-19 pandemic.

Variable	First Wave (*N* = 1644)	Second Wave (*N* = 720)	*p*-Value
Comorbid chronic disease			
No	1306 (79.4%)	560 (77.78%)	0.362
Yes	338 (20.6%)	160 (22.22%)	
On treatment due to any disease			
No	1072 (65.2%)	440 (61.11%)	0.056
Yes	572 (34.8%)	280 (38.89%)	
In quarantine			
No	1590 (96.7%)	598 (83.06%)	<0.001
Yes	54 (3.3%)	122 (16.94%)	
Friends/family in quarantine			
No	1351 (82.2%)	336 (46.67%)	<0.001
Yes	293 (17.8%)	384 (53.33%)	
History of contact with infected with COVID-19			
No	1615 (98.2%)	473 (65.69%)	<0.001
Yes	29 (1.8%)	247 (34.31%)	
Diagnosed with COVID-19			
No	1638 (99.6%)	674 (93.61%)	<0.001
Yes	6 (0.4%)	46 (6.39%)	
Friends/family infected with COVID-19			
No	1544 (93.9%)	260 (36.11%)	<0.001
Yes	100 (6.1%)	460 (63.89%)	
Friends/family died of COVID-19			
No	1620 (98.5%)	649 (90.14%)	<0.001
Yes	24 (1.5%)	71 (9.86%)	

**Table A3:** Psychological characteristics of the study group during the first and second wave of the COVID-19 pandemic.

Variable	First Wave (*N* = 1644)	Second Wave (*N* = 720)	*p*-Value
Under psychiatric/psychological care during COVID-19 pandemic			
No	1537 (93.5%)	616 (85.56%)	<0.001
Yes	107 (6.5%)	104 (14.44%)	
On sedatives during COVID-19 pandemic			
No	1520 (92.5%)	668 (92.78%)	0.785
Yes	124 (7.5%)	52 (7.22%)	
Fear of infection with coronavirus has negative impact on my mental health			
Strongly agree	125 (7.6%)	61 (8.47%)	
Agree	410 (24.9%)	180 (25.00%)	
Undecided	382 (23.2%)	134 (18.61%)	0.139
Disagree	393 (23.9%)	189 (26.25%)	
Strongly disagree	334 (20.3%)	156 (21.67%)	
Fear of heath condition of the loved ones is a source of stress and depressed mood			
Strongly agree	302 (18.4%)	179 (24.86%)	
Agree	642 (39.1%)	290 (40.28%)	0.003
Undecided	234 (14.2%)	96 (13.33%)	
Disagree	305 (18.6%)	93 (12.92%)	
Strongly disagree	161 (9.8%)	62 (8.61%)	
Following the media reports is a source of a significant deterioration of my mental state			
Strongly agree	344 (20.9%)	189 (26.25%)	
Agree	461 (28.0%)	209 (29.03%)	0.017
Undecided	307 (18.7%)	106 (14.72%)	
Disagree	295 (17.9%)	116 (16.11%)	
Strongly disagree	237 (14.4%)	100 (13.89%)	
Perceived loneliness caused by isolation from the world/loved ones			
Strongly agree	528 (32.1%)	223(30.97%)	
Agree	191 (11.6%)	223 (30.97%)	
Undecided	176 (10.7%)	91 (12.64%)	<0.001
Disagree	191 (11.6%)	107 (14.86%)	
Strongly disagree	187 (11.4%)	76 (10.56%)	
More frequent use of alcohol/cigarettes cause by pandemic			
Strongly agree	165 (10.0%)	49 (6.81%)	
Agree	235 (14.3%)	105 (14.58%)	0.118
Undecided	154 (9.4%)	61 (8.47%)	
Disagree	257 (15.6%)	121 (16.81%)	
Strongly disagree	833 (50.7%)	384 (53.33%)	

**Table A4:** Correlations between BDI, FSFI and COVID-19-related characteristics.

Variable	Variable	I Wave		II Wave		Fisher’s z
		Correlation Coef.	*p* Value	Correlation Coef.	*p* Value	
BDI	FSFI	−0.3261	<0.001	−0.2769	<0.001	0.2267
FSFI	Age	0.04983	0.0434	0.0662	0.076	0.5573
	In quarantine	0.02175	0.3782			
	Diagnosed with COVID-19	−0.01121	0.6496			
	Comorbid chronic disease	−0.08747	<0.001			
	Fear of infection	−0.08848	<0.001	−0.1290	0.01	0.3597
	Fear of heath condition	−0.1016	<0.001	−0.0824	0.027	0.6654
	Following the media	−0.1046	<0.001	−0.0426	<0.084	0.1679
	Perceived loneliness	−0.1527	<0.001	−0.1141	<0.01	0.38
	More frequent use of alcohol/cigarettes	−0.03532	0.1523	0.0064	0.864	0.3515
BDI	Age	−0.3261	<0.001	−0.1970	<0.001	<0.001
	In quarantine	−0.02053	0.4055			
	Diagnosed with COVID-19	0.01882	0.4456			
	Comorbid chronic disease	0.05604	0.0231			
	Fear of infection	0.2936	<0.001	0.2556	<0.001	0.3586
	Fear of heath condition	0.3047	<0.001	0.2502	<0.001	0.2952
	Following the media	0.2738	<0.001	0.2260	<0.001	0.262
	Perceived loneliness	0.3923	<0.001	0.3083	<0.001	0.324
	More frequent use of alcohol/cigarettes	0.2308	<0.001	0.2744	<0.001	0.2982

BDI, Beck Depression Inventory; FSFI, Female Sexual Function Index.

There was a significant age difference (median = 22 vs. median = 23; *p* < 0.001). However, the numerical difference was one year, which was not clinically significant. The histograms show that groups were similarly distributed and all women were at reproductive age.

We estimated the sample size using the G* power package. A priori, we set the Mann–Whitney U test (two groups) with an average effect size of d = 0.50, a power (1-β) of 0.95, a probability level of α = 0.05 and an allocation ratio of 1/3. With these assumptions, the total study group should have included 244 (61/181) participants. The total study group was 2364 participants (about 10 times more than required), which means it was overpowered. The reason for this is related to the fact that with larger samples, it is easier to show statistical differences between study groups. In this case, those differences were not clinically significant.
